# Neuro-oncology family caregivers’ view on keeping track of care issues using eHealth systems: it’s a question of time

**DOI:** 10.1007/s11060-017-2504-y

**Published:** 2017-05-26

**Authors:** Florien W. Boele, Cornelia F. van Uden-Kraan, Karen Hilverda, Jason Weimer, Heidi S. Donovan, Jan Drappatz, Frank S. Lieberman, Irma Verdonck-de Leeuw, Paula R. Sherwood

**Affiliations:** 1 School of Nursing, University of Pittsburgh, 336 Victoria Bldg, Pittsburgh, PA 15261 USA; 2 Leeds Institute for Cancer and Pathology, St James’s Hospital, University of Leeds, Beckett Street, Leeds, LS9 7TF UK; 3 Leeds Institute of Health Sciences, University of Leeds, Worsley Building, Clarendon Way, Leeds, LS2 9NL UK; 4 Department of Clinical, Neuro and Developmental Psychology, VU University, Van der Boechorststraat 1-3, 1081BT Amsterdam, The Netherlands; 5 Department of Medical Psychology, VU University Medical Center, PO Box 7057, 1007MB Amsterdam, The Netherlands; 6 Division of Neuro-Oncology, Hillman Cancer Center, University of Pittsburgh Medical Center, 5115 Centre Avenue, Pittsburgh, PA 15232 USA

**Keywords:** Family caregiver, Brain tumor, Neuro-oncology, Supportive care, eHealth

## Abstract

Primary brain tumors (PBTs) are rare but have a great impact on both patient and family caregiver wellbeing. Supporting caregivers can help them to continue their caregiving activities to maintain the patients’ best possible level of quality of life. Efforts to improve PBT caregiver wellbeing should take into account country- or culture-specific differences in care issues and supportive care needs to serve larger caregiver groups. We aimed to explore PBT caregivers’ satisfaction with the current supportive care provision, as well as their thoughts on monitoring their care issues with both paper-based and digital instruments. Twelve PBT caregivers were interviewed in the United States. The semi-structured interviews were transcribed verbatim and analyzed by two coders independently. Data were combined with those collected in the Netherlands, following similar methodology (*N* = 15). We found that PBT caregivers utilize both formal and informal support services, but that those who experience more care issues would prefer more support, particularly in the early disease phase. Keeping track of care issues was thought to provide more insight into unmet needs and help them find professional help, but it requires investment of time and takes discipline. Caregivers preferred a brief and easy-to-use ‘blended care’ instrument that combines digital monitoring with personal feedback. The present study shows that the preferences of family caregivers in neuro-oncology toward keeping track of care issues are likely not heavily influenced by country- or culture-specific differences. The development of any instrument thus has the potential to benefit a large group of family caregivers.

## Introduction

The diagnosis and treatment of a primary brain tumor (PBT) can have a devastating effect on the patient and their family. With the prognosis and treatment options being highly dependent on the tumor type and grade, and many tumors eventually recurring or progressing [1], dealing with uncertainty of the future is a major issue for most patients [2]. Moreover, disease-specific symptom burden can include debilitating physical and neurological sequelae such as paresis, sensory loss, visual-perceptual deficits, cognitive deficits, and seizures [3, 4]. Changes in personality and behavior, fatigue and mood issues also occur frequently [5, 6]. Together, these symptoms can hinder patients’ ability to function independently and influence their quality of life (QOL) as well as the QOL of their family members.

Family members and close friends commonly provide patients with requisite physical and emotional support, and are therefore called ‘family caregivers’. This can be a rewarding task, but many family caregivers also report considerable burden and distress as a result of providing care [7]. Not only may the psychological and behavioral responses to caregiving influence caregivers’ emotional and physical health [8], it may also influence the quality of care delivered to the patient at home. Professional support in meeting the needs of family caregivers to decrease their distress, is imperative to enable them to continue their caregiving activities and maintain the best possible level of patients’ wellbeing.

Frequent monitoring with patient reported outcomes (PROs) to track needs over time, paired with feedback and followed up with supportive care as needed has been suggested as a possible way to meet patients’ needs [9]. The same principle may be applied to caregivers in meeting their own needs. This monitoring may be introduced into clinical practice through administering paper questionnaires, or by means of computerized or web-based (eHealth) applications. With the growing popularity of eHealth and ongoing advances in technology, applications are sometimes developed before consulting end users’ opinions on their preferences [10]. This can harm the effectiveness and uptake of eHealth solutions and indeed, it has been suggested that engaging the family caregivers as end users in the development of any pathway to resolve supportive care needs is vital to improve its usefulness in practice [10, 11].

In our previous report on the attitudes and preferences of Dutch glioma patients and their family caregivers regarding monitoring symptoms, distress and QOL issues, we found that although the preferred method for monitoring issues is highly personal, a combination of online and personal care (‘blended care’) was advised [12]. Importantly, we found that family caregivers viewed monitoring symptoms, distress and QOL (collectively called ‘care issues’ hereafter) more favorably than patients did. Details on patients’ and caregivers’ perceived (dis)advantages for keeping track of symptoms, distress and QOL issues were provided and pros and cons for both paper-based and computer-based methods were discussed. However, our previous report lacks details needed to develop or adjust monitoring systems to best meet PBT caregivers’ preferences and needs. Moreover, there may be country or culture specific differences in the expression of distress, QOL, or supportive care needs [13], as well as differences in the organization of health care and accessibility of supportive care between different countries. As PBTs are rare and the needs of the family caregivers do not always overlap with those of other oncological caregiving populations, it is vital that any instrument developed has the potential to benefit as many PBT caregivers as possible.

Therefore, we now add data from a sample of neuro-oncology caregivers from the United States and reexamine the transcripts from the Dutch caregiver interviews. Specifically, the purpose of this study is to investigate in detail how to adjust monitoring care issues to the preferences of family caregivers in neuro-oncology in general by focusing on: (1) experiences with current supportive care provision; (2) caregivers’ ideas on how to make monitoring care issues more acceptable; (3) and their preferences, as well as the perceived (dis)advantages of paper-based and digital monitoring instruments. Recommendations will be made which can contribute to the future development of instruments to monitor PBT caregiver issues.

## Methods

### Sample and procedure

Caregivers were recruited at the outpatient department of a NCI-designated Cancer Center between August and November 2015. Caregivers were eligible if they (1) were >18 years old; (2) were the primary family caregiver of a patient diagnosed with a PBT (any type). Caregivers could not participate if they did not provide informed consent or if they had insufficient understanding of the English language to provide consent and/or complete the interview. Additionally, caregivers could not participate if the patient did not provide informed consent for the caregiver to be interviewed, or if they lived further than a 3 h drive from the Cancer Center and were unable to be interviewed at the hospital. The treating physician, physician’s assistant, or nurse practitioner introduced potential participants to the study and obtained permission to be approached by a member of the research team, who then contacted them regarding their willingness to participate. Purposive sampling was done to ensure participants represented the general caregiving population.

Interviews were semi-structured and lasted for about 60 min, taking place either in the hospital or at a location of the participant’s preference. Interviews were performed until data saturation occurred, meaning that no new information was generated by the last interview(s) [14]. All interviews were recorded. Data were combined with those collected during a previous study performed in the Netherlands, following similar methodology [12]. All participants signed written, informed consent forms and the study protocols were approved by the University of Pittsburgh (PRO15040347) and the Institutional Review Board of the VU University Medical Center (protocol number 13/309).

### Interviews

Interviews were introduced to participants by explaining that we were interested in their care issues, their experiences with supportive care, and their thoughts and ideas regarding different methods to keep track of their care issues over time, so that issues may be recognized and information and support may be accessed in a timely manner. The same semi-structured interview schedule from our previous publication was used [12]. To summarize, the main topics were (1) experiences with supportive care provision and unmet supportive care needs; (2) thoughts and suggestions regarding keeping track of care issues, including frequency, timing and format; (3) attitudes toward and conditions for use of different paper-based (the Distress Thermometer [15]) and eHealth instruments (Oncoquest [16] and Oncokompas [17]) to track care issues over time. Although these instruments are all patient-oriented, we felt these were suitable examples to show caregivers because they rely on technology in various degrees: from no technology being used with the distress thermometer (DT), to Oncoquest (OQ) which is part computer-based and part comprised of personal contact, to Oncokompas (OK) which is completely computer-based. Also, the degree to which health care professionals would be involved varies: the DT and OQ instruments would ideally be fed back by a nurse, who would then initiate referral if needed, whereas OK is a self-management tool. Participants were shown printed pictures and screen shots accompanied by English translations where appropriate, and detailed verbal explanation of the instruments was provided. Prompts were offered if open-ended questions elicited little response. Interviews were performed by a research psychologist (FWB)—or, in one case, by a PhD-student with experience in interviewing for qualitative analysis and supervised by FWB (NSK; acknowledgments).

### Data analysis and reporting

The interviews were transcribed verbatim. Thematic data analysis [18, 19], similar to the methods used in our previous publication [12], was conducted by two coders (PRS and FWB). The coders read the transcripts several times to familiarize themselves with the content, and highlighted sections of the transcripts that were related to the research objectives and independently selected and coded these into key issues and themes. The coders met frequently to discuss their findings, refine the key issues and themes, and resolve possible differences until consensus was reached. If needed, a third researcher (CFU) was available to guide this process. Finally, one coder (FWB) examined all transcripts again, including the transcripts from the Dutch interviews, to ensure that the analytical process was robust. Where applicable differences between the US and Dutch caregiver samples are highlighted in the Results. The consolidated criteria for reporting qualitative research (COREQ) [20] were used in this report. All quotes provided were anonymized.

## Results

### Participants

Twenty-three caregivers were invited, of whom 12 agreed to participate. Reasons for non-participation were: not interested (*N* = 5); too busy (*N* = 2); unable to reach by phone to schedule interview (*N* = 2); declined home visit and interview could not be scheduled in the hospital due to time constraints (*N* = 1). Data saturation was reached with the 12th participant. The sample is representative of caregivers of patients with a wide range of neuro-oncological diagnoses across different disease stages; see Table 1 for characteristics of caregivers from the present sample and the previously collected Dutch sample.

**Table 1:** Demographic characteristics of participants

	US sample (*N* = 12)	Dutch sample (*N* = 15)
Age		
30–40	2 (16.7%)	1 (6.7%)
40–50	2 (16.7%)	3 (20%)
50–60	3 (25%)	9 (60%)
60–70	4 (33.3%)	1 (6.7%)
70–80	1 (8.3%)	1 (6.7%)
Sex		
Male	6 (50%)	2 (13.3%)
Female	6 (50%)	13 (86.7%)
Relationship to patient		
Spouse	10 (83.3%)	15 (100%)
Parent	1 (8.3%)	0 (0%)
Cousin	1 (8.3%)	0 (0%)
Diagnosis of patient		
Oligodendroglioma grade II	2 (16.7%)	3 (20%)
Astrocytoma grade II	1 (8.3%)	1 (6.7%)
Oligodendroglioma grade III	0 (0%)	3 (20%)
Astrocytoma grade III	1 (8.3%)	2 (13.3%)
Glioblastoma grade IV	6 (50%)	7 (46.7%)
Medulloblastoma	1 (8.3%)	0 (0%)
Meningioma	1 (8.3%)	0 (0%)
Time since diagnosis		
<1 year	1 (8.3%)	5 (33.3%)
1–2 years	5 (41.7%)	1 (6.7%)
2–3 years	2 (16.7%)	3 (20%)
3–4 years	1 (8.3%)	0 (0%)
4–6 years	0 (0%)	1 (6.7%)
>6 years	3 (25%)	5 (33.3%)
Disease status		
Stable disease	2 (16.7%)	10 (66.6%)
Disease progression (suspected)	4 (33.3%)	2 (13.3%)
Under treatment	6 (50%)	3 (20%)

### Experiences with supportive care for caregivers

The support family caregivers reported to have received or have been offered can be categorized into:

(1) Formal support from health care and religious services; (2) informal support from friends, family and community members; and (3) support from work, see Table 2. Formal support services included support from specialist nurses either through the neuro-oncology clinic, psychiatry or primary care physician’s office; social work or psychology services; support groups for caregivers; practical support such as meals on wheels; and medication (e.g. antidepressants, sleep medication). US caregivers specifically frequently mention religious services such as pastoral counseling, and only Dutch caregivers report having received legal advice, help with completing tax forms in light of their special circumstances and transportation services to and from the hospital.

**Table 2:** Caregivers’ experiences with supportive care

Topics	Key issues	Themes	Participant groups
Formal support	Emotional support	Nurse(-specialist)	Both groups
		Psychologist	Both groups
		Social worker	Both groups
		Peer support group	Both groups
		Medication (e.g., antidepressants, sleeping pills)	Both groups
		Religious services	US group
	Practical support	Nurse(-specialist)	Both groups
		Meals on wheels	US group
		Transportation service to/from the hospital	Dutch group
		Legal advice, e.g., preparing a will	Dutch group
		Help with completing tax forms	Dutch group
	Needs screening in clinic	Questionnaires in clinic without feedback or referral	Both groups
Informal support	Social support	Family and friends being there in the hospital	Both groups
		Supporting each other through tough times	Both groups
		Family and friends watching the patient when the caregiver’s away	US group
		Family or friends setting up Facebook account to provide support	Both groups
		Family member coming back to live at home and provide support	US group
		Community members praying for patient and caregiver	US group
	Practical support	Making dinner, bringing food over to the house	US group
		Getting groceries	US group
		Helping with household chores	US group
		Helping the patient get ready for the day	US group
		Providing a wake-up call service	US group
		Friend with medical background would answer questions	US group
	Financial support	Taking up a collection to support a housekeeper	US group
		Raising money to help pay for medical bills	US group
		Raising money to provide patient and caregiver with a dinner date	US group
Support from work	Flexible work hours	Caregiver is allowed to leave work early	Both groups
		Caregiver can take leave when needed	Both groups
		Caregiver can cut back on hours, or work more depending on the situation	US group
	Flexible work place	Caregiver can work from home	Both groups
	Flexible responsibilities	The duties at work are adjusted because of the mental state of the caregiver	Dutch group
	Work-based formal health services	Counselling service for mental support	Dutch group
		Company doctor	Dutch group

Examples of informal support provided include *social support* such as being there in the hospital, supporting each other through tough times; *practical support* such as people coming over to bring dinner, deliver groceries, or helping out with other chores; and *financial support* such as helping to pay for medical bills or housekeeping. Particularly practical and financial support were mentioned more often by US caregivers, e.g.:

‘They took up a collection through a variety of different things. They had a housekeeper come in and clean our house for 6 months. For 6 months! They paid for it. […] It was tremendous.’

Husband of GBM patient who is currently under treatment

Of note, two caregivers expressed that too much informal involvement can be a burden as well, as continually having to explain to family and friends that the patient is not well despite their healthy appearance and the lack of time to be together as a couple can be difficult. Under support from work, many caregivers reported their employers had been very understanding and allowed them to cut back on hours and/or increase hours as needed, and some mention having been given the option to work from home.

In a few cases, questionnaires were used in the clinic to screen for needs. However, none reported to have received any feedback and if needs were identified, there was no further follow-up or referral to professional services. When asked if they felt the supportive care offered was sufficient, caregivers with few care issues were generally satisfied while others indicated they would have liked more information on how to deal with the patient’s symptoms and prognosis, as well as more practical, financial and social support especially early in the disease trajectory.

‘Oh I think we need something! We’re not professionals, we don’t know what we’re doing. […] Are we making it better, are we making it worse? We don’t know, we’ve not been in this situation before.’

Cousin of meningioma patient with suspected disease progression

Common barriers to supportive care services were: support was only offered if actively requested; support was not offered when caregivers and patients appeared to have a good informal support system; not knowing where to find local services with expertise in neuro-oncology; the support offered was not a good fit given specific neuro-oncological issues; not qualifying for support when issues were not grave enough; and costs and lack of health insurance coverage. Some caregivers said they had developed their own coping mechanisms such as exercising regularly, thinking positively, counting blessings, and living life 1 day at a time.

### Attitudes toward keeping track of care issues over time

#### General attitude

Caregivers generated several possible advantages of keeping track of care issues over time. It could: help them gain insight into their own needs and growing burden, so that they might get help in time; give them a sense of being in control of the situation; help them be more perceptive of changes in the patient’s symptoms; and some believed that keeping track of issues could help them process grief. Disadvantages were also mentioned. Some caregivers said they do not like keeping track of things per se and many thought that just keeping track would not benefit them. Another disadvantage frequently mentioned by caregivers is that it takes time and requires discipline:

‘I don’t know if there’s a disadvantage, other than the fact that it’s time consuming. It takes time to do that and it takes discipline. You have to have discipline to sit down and journal. […] And this, I look at this company, forget whether it’s health related, if we ask people to journal, so they knew what they were spending their time on and what was most important to them during the day, we probably get about 5% compliance. It’s just, that’s a hard thing to do on a regular basis.’

Husband of GBM patient who is currently under treatment

Moreover, a few caregivers indicated that it could be difficult to be confronted with their situation frequently. Some caregivers clearly indicated that keeping track of their issues and needs would not be for them. However many were uncertain about their future needs and care issues and emphasized that keeping track of care issues systematically would be most useful around the time when many needs are present. For most, this included the periods immediately post-diagnosis and around disease progression.

#### Frequency and timing

Preferences for the frequency of monitoring care issues varied widely, ranging from once a week to once every 18 months. Many indicated that their preferred frequency would depend on the needs they experience and that it would therefore be good to start monitoring shortly after the patient is diagnosed. In terms of timing and setting, most caregivers indicated it would be best for them to complete questions at home, before visiting the hospital. This would allow more time under less stressful circumstances, and lead to more honest answers because of better privacy. Moreover, as not all caregivers were always able to accompany the patient to clinic, this would allow them to keep track of their care issues and needs more frequently. Others however, felt their often long waiting time in the hospital could be put to good use by completing questions then and there.

#### Format

Presented with different options to keep track of their issues over time, only a few caregivers preferred a yes/no type checklist as it is easy to complete and unambiguous, while the majority of caregivers believed a multi-item scale would reflect their situation better:

‘This is just a concrete yes or no. And it could be like oh I’m having a little bit of a problem with child care or I’m in desperate need of help. And this kind of tells you how big of a problem each individual thing is.’

Wife of oligodendroglioma grade II patient with suspected disease progression

A few suggested that a diary form would be best, or alternatively to allow room for free text remarks. Some emphasized that any questionnaire should be as concise as possible to reduce burden. Although caregivers said a paper-based checklist (DT) would be quick and easy to complete, includes relevant topics and would give a good overview of the issues that are present in one glance, they were unsure how they would benefit from completing the checklist:

‘Will it provide some way to foresee down times and be able to… You know, help reduce the stress to look for a way to kinda work around it? I don’t know, I’m not sure that I could… That it would have like a lot more value than ‘hmm that’s interesting’. Would it really, would I find a way to make it a less stressful time? You know based on that? I’m not sure it would.’

Husband of patient with medulloblastoma who is receiving treatment

Caregivers indicated that personal feedback from a health care professional would help, but some participants felt this would not be realistically achievable in clinical practice. Although many caregivers mentioned that an eHealth monitoring instrument that comprises a touch-screen system based in the clinic (OQ) would not be ideal as it is not mobile, they did feel completing the questions in this digital manner would be easier and faster than on paper, and better for the environment. Several caregivers said the fully digital option comprising a home-based monitoring instrument (OK) could serve as a guide to supportive care options as the resources it generates are suggested by a trusted source (i.e., the hospital). The opportunity to complete the questions at home and the flexibility in terms of timing were mentioned as important advantages:

‘I like it ‘cause you could do it at your house, whenever you want to, privacy of your home, be comfortable, and you know. It’d just be you and the computer. I like that.’

Wife of patient with astrocytoma grade II who is under treatment

Negative points raised regarding the fully digital option (OK) were the expected high investment of time, an expected need for reminders, and the lack of personal contact.

Table 3 shows caregivers’ preferences regarding paper-based and eHealth monitoring systems in general. EHealth monitoring instruments were regarded as having high accuracy and comfortable levels of privacy when completing personal questions. The anonymity that comes with computer use was seen as an advantage especially when experiencing high levels of distress, as it can make expressing their true feelings easier. Important tips for the development of eHealth instruments provided by Dutch caregivers were: to limit the information generated to avoid an information overload, and to automatically save completed questions to reduce frustration if the system crashes (see Table 3). Several caregivers indicated that mobile solutions that allow use on tablets or smartphones would be superior to desktop only instruments. Stand-alone eHealth instruments such as OK were regarded as sufficient when few needs or issues are present, and adding the option to contact a health care professional either digitally, by phone or in person if needed was suggested by several caregivers.

**Table 3:** Caregivers’ preferences for paper-based, digital or blended care monitoring instruments

Topics	Key issues	Themes	Participant groups
Type of monitoring instrument	Preference for…		
	Paper-based with personal feedback	It is quick and easy to complete	Both groups
		Would provide a quick overview of the needs present	Dutch group
		Not interested in using computers	US group
	Fully digital	Can be completed at home, which is comfortable, allows privacy, and time to focus	Both groups
		Feedback on needs and issues is provided instantly	Both groups
		Professional advice for referral to supportive care is provided instantly	Both groups
		The data and feedback are trusted to be accurate	Both groups
		It is always accessible, flexible	Both groups
		Allows you to go back to the advice to read at leisure	Dutch group
		Requires less action than other methods—you just complete questionnaires once and it’s done	US group
		Saves paper, probably cheaper in long run	US group
	Blended care (i.e. digital with personal contact)	Digital option would suffice when there is no great need for support; adding personal contact would only be required when needs arise	Both groups
		Might suffice to just add the option to actively contact someone if need arises	Both groups
		Personal contact can help verbalize issues	US group
		Personal contact provides extra support—talking can help to relieve stress	US group
		It is more interactive	Both groups
Reasons for (non)use of any instrument	Perceived as beneficial	Most likely to use if there are needs present	Both groups
		Would only continue to use if there is perceived benefit	Both groups
	Clarity	Should be clear and easy-to-use	Both groups
	Investment of time	The likeliness to use an instrument is dependent on the time it takes	Both groups
		Should not take more than 10 min to complete questions	US group
	Available resources	Would only be useful if supportive care is actually available to them	Both groups
		Resources should fit neuro-oncology specific needs	Both groups
		Guide toward good information, good supportive care options, and good stress-relieve techniques	US group
	Recommendation by treatment team	Would not seek out a monitoring instrument without recommendation by treatment team	Both groups
		Would help recognize that it is not just another internet resource	Dutch group
		Recommendation would help as incentive to start only	Both groups
	Continued use	An extra incentive is necessary for people to continue to keep track: money or a personal coach	US group
		Reminders are needed to continue use, especially when things are going well	Both groups
		A personal (face-to-face) reminder for use can help	US group
Reasons for (non) use of digital instruments	Anonymity and privacy	Easier to be honest about your feelings when you feel like you’re by yourself	Both groups
		Easier to answer questions presented digitally when stressed	US group
		Privacy is a prerequisite; can be difficult to answer questions when patient is looking over your shoulder	Dutch group
	Accuracy of digital data	Data is accurate and easy to quantify for clinicians/researchers	US group
	Limit information	Presenting one question at a time can help to retain focus	Dutch group
		Be wary of information overload on computer systems	Dutch group
	Automatic saving	Can help to save answers frequently and automatically	Dutch group
	Computer and internet access	Would not use the instrument on a public computer	US group
		Slow computer would discourage use	US group
	Mobile solutions	Mobile solutions might be better than PC-based ones	Both groups
		Apps are always available	US group
	Computer literacy	Good to very good computer literacy	Both groups
		Can use the computer for ‘simple things’, including google, home shopping and banking	Both groups
		Can use the computer but is not very used to it	US group
		Not one to sit in front of a computer	Both groups
		Not computer literate, can’t even turn it on	US group
		Most elderly people can also use the computer nowadays	US group
		Can be difficult for older people	US group
	Alternatives	For those not computer literate, a paper questionnaire might be used as an alternative	US group

‘So I think if you maybe had the answers like the short answers and then give the opportunity to say tell me what you meant by this. And then it will all come out. To give them the opportunity to not be hit by it all of a sudden.’

Wife of oligodendroglioma grade II patient with suspected disease progression

Almost all caregivers felt able to use an eHealth instrument and most, but not all, caregivers opted for either a fully digital instrument or a combination of a digital instrument with personal feedback. A few caregivers, on the other hand, preferred a paper-based checklist with personal feedback instead, and one caregiver suggested to make all options available so that all personal preferences may be reflected.

#### Conditions for use

Caregivers made several suggestions for the development of any monitoring instrument, see Table 3. Many said they would not make use of an instrument unless they perceive benefits for themselves; this could be related to the needs present, or to the options for supportive care that are available and affordable. Participants said that any needs assessment should be quick to complete, preferably under 10 min, and be clear and easy to use. Furthermore they indicated that the resources that are referred to, should be tailored specifically to the neuro-oncology situation. Many believed recommendation by a trusted source such as the treatment team would help them to start using a monitoring instrument but it was expected that an incentive (either the presence of supportive care needs, a small monetary reward, or frequent reminders) would be needed to ensure continued use:

‘I think some people would say yeah that’s a good thing, the doctor’s telling me that. There’s other people who’ll forget about it once they’re in the car. There has to be constant reminders. […] Just an idea, some kind of marketing pushout to people on a regular basis.’

Husband of patient with medulloblastoma who is under treatment

Other tips generated by US caregivers include that any computerized option should be flexible enough to use on different personal devices as public computers were generally not trusted, and should not take up too much of the device’s working memory as a slow computer would put them off.

## Discussion

This qualitative study is an extension of our earlier report on PBT caregivers’ attitudes and preferences toward monitoring care issues [12]. Although PBT caregivers reported to have utilized a range of different formal and informal support services, those who experienced more care issues indicated to have preferred more support. Appropriate and available supportive care services are, however, difficult for caregivers to find, underlining the important contribution to signposting a monitoring instrument could have. Regarding keeping track of care issues over time, the present study findings from the interviews with family caregivers from the US, support and enrich the results from our previous publication. Similar advantages and disadvantages of keeping track of care issues were mentioned. On the one hand, PBT caregivers feel it can provide more insight into one’s own needs and through this avenue, it could help them find their way to supportive care. On the other hand, it takes valuable time and requires discipline, and it can be difficult to face seeing their care issues increase over time. Importantly, PBT caregivers would only find it useful to keep track of their care issues when there are needs present. They commonly felt a short and easy-to-use ‘blended care’ instrument, that combines digital monitoring with some kind of personal feedback, was most appropriate for them. Furthermore, most caregivers would prefer to complete questions at home, stating that the clinic visit is very stressful and not a good indicator of overall stress. Several useful tips were generated by PBT caregivers that could facilitate instrument development.

The preference for a blended care option corroborated findings from studies among cancer patients in general [21] and neuro-oncology patient/caregiver populations in particular [22]. Cancer survivors (*N* = 212) viewed eHealth applications favorably, with 48% indicating that they would find the option to self-monitor their side-effects and symptoms attractive [21]. A feasibility study testing a Danish brain tumor website among brain tumor patients and caregivers furthermore showed that especially caregivers appreciated the option to contact health care professionals online [22]. However, our report focuses on caregivers’ preferences for keeping track of their own care issues over time—which is, indeed, a relatively unexplored area [23, 24].

Our findings confirm that the preferences and attitudes of family caregivers in neuro-oncology are likely not heavily influenced by country- or culture-specific differences. Of course, differences in the experiences and satisfaction with supportive care have been observed between US and Dutch PBT caregivers. Any caregiver monitoring instrument could take these differences into account to ensure advice on supportive care services meets the needs. Religion and religious communities as well as practical and financial aid from community members played a larger role in the support system of US caregivers, whereas Dutch caregivers more frequently reported having received formal support from the work place, legal advice, and transportation services. This reflects differences between the two countries in terms of health care organization [25–27] and (religious) culture [28, 29]. However, the experienced care issues and unmet supportive care needs expressed were similar. Therefore, the development of any instrument has the potential to benefit a large group of family caregivers. It would however, remain interesting to interview family caregivers from less developed countries or from non-Western cultures as well, to see if results hold.

A limitation of this study is that not all interviews were performed by the same person, which could potentially have influenced study outcomes. Rather, a research psychologist involved in both research groups (FB) completed (or in one case, supervised) the US interviews whereas the Dutch interviews were conducted by a trained and registered clinical psychologist (KH). To minimize the possible influence of this change in interviewers, the semi-structured interview schedule was translated directly from Dutch to English and the same examples of monitoring instruments were used. For practical reasons we chose to only recruit participants who could either be interviewed in the clinic or lived within a 3 h drive from Pittsburgh. This radius was also chosen because it includes both urban and rural areas and has resulted in a good and varied representation of participants in our previous studies. The US and Dutch samples showed some differences in composition, with the US group including a greater variety of tumor types and the Dutch sample including fewer male caregivers. Combined, these groups are a good representation of the population, however the differences between the groups might have contributed to some of the differences described in this report. Where our previous report focused on participants’ thoughts on specific existing monitoring instruments, here, we used the same instruments as examples but aimed to provide broader recommendations for future instrument development. This is necessary as at present, there is no (neuro-oncology) caregiver-specific monitoring instrument available in clinical practice. Although several interventions have been performed to try and provide support for family caregivers in neuro-oncology, some utilizing eHealth or telehealth solutions [30], none so far include the option to keep track of care issues over time to only provide supportive care as needed. Caregivers indicated they would only want to use a monitoring instrument when they experience care issues. Predicting when issues will arise is, however, very difficult for both health care professionals and caregivers, so any monitoring instrument would have to be as short as possible to minimize the added burden and improve compliance. The suggestions and ideas generated by caregivers can serve as pointers in adapting and optimizing any evidence-based instrument for use by family caregivers in neuro-oncology.

To summarize, we found that two separate samples of family caregivers in neuro-oncology prefer a blended care option that combines digital monitoring with personal contact. Adapting and implementing a blended care instrument to keep track of issues and supportive care needs over time has the potential to benefit large groups of family caregivers in neuro-oncology.
